# Gut microbiome dysbiosis as a potential biomarker for liver metabolic disorders in in neonatal hemolytic jaundice

**DOI:** 10.1186/s12887-025-05692-8

**Published:** 2025-04-29

**Authors:** Jin Huang, Bi Zhou, Feng Zhu, Ying Li, Yingying Li, Rui Zhang, Jingling Zhang, Lili Wang

**Affiliations:** 1 Department of Pediatrics, The First Affiliated Hospital of Anhui Medical University, Hefei, Anhui Province 230000 China; 2 Department of Pediatrics, Suzhou Hospital of Anhui Medical University (Suzhou Municipal Hospital of Anhui Province), Suzhou, Anhui Province 234000 China

**Keywords:** Hemolytic jaundice, Neonate, Gut microbiota, *Enterobacter*, 16S rRNA

## Abstract

**Background:**

This study aims to reveal the composition and features of the gut microbiota in neonatal hemolytic jaundice, potentially identifying biomarkers for the diagnosis of this condition.

**Methods:**

A total of 62 neonates with hemolytic jaundice and 20 healthy neonates were ultimately enrolled in the study. Clinical data and fecal samples from these infants were collected separately. The composition and features of the gut microbiota were analyzed using 16S rRNA high-throughput sequencing technology. Alpha and Beta diversity analyses were conducted to elucidate the differences in gut microbiota composition. Additionally, LEfSe analysis was employed to identify differential microorganisms. Finally, PICRUSt2, metagenomeSeq, and BugBase software were utilized to investigate the phenotypic and functional differences in the gut microbiota.

**Results:**

Beta diversity analysis revealed significant differences in the composition of gut microbiota. LEfSe analysis demonstrated a significant increase in the relative abundance of *Enterobacter* in neonatal hemolytic jaundice. Furthermore, METACYC metabolic pathway analysis based on PICRUSt2 indicated a notable elevation in liver-related metabolic pathways in neonatal hemolytic jaundice. The metabolic analysis of differential bacterial genera revealed that *Enterobacter* secretes a wide array of enzymes, including oxidases, oxidoreductases, transferases, hydrolases, isomerases, and lyases. Notably, these enzymes are responsible for altering the liver metabolic pathways in neonates with hemolytic jaundice.

**Conclusions:**

*Enterobacter* is linked to multiple metabolic pathways in the liver via the secretion of numerous enzymes along the gut-liver axis metabolic pathway. This interaction indirectly reflects the metabolic status and disease progression in neonatal hemolytic jaundice. Consequently, *Enterobacter* may serve as a potential diagnostic marker of the gut microbiota for assessing liver metabolic disorders associated with hemolytic jaundice.

## Introduction

Neonatal jaundice is a prevalent condition during the neonatal period, characterized by an abnormal elevation of bilirubin levels in the body, which results in the yellowing of the skin, mucous membranes, and sclera in newborns. This condition can be categorized into physiological and pathological jaundice [1, 2]. Physiological jaundice is linked to the underdeveloped physiological traits of newborns and typically does not result in significant complications [3]. In contrast, pathological jaundice is characterized by an abnormal elevation in serum bilirubin levels, which can be caused by various factors, including neonatal infections, hemolysis, and inadequate breastfeeding. Notably, hemolytic jaundice develops most rapidly and is the most severe form [4, 5].

Hemolysis is a pathological process marked by the premature destruction of red blood cells (RBCs) in circulation, leading to hemoglobin release into the bloodstream and triggering a range of clinical symptoms [6]. Under normal physiological conditions, aged RBCs are efficiently recognized and cleared by macrophages in the spleen and liver. However, when RBC destruction is accelerated due to factors such as genetic defects, immune-mediated attacks, mechanical injuries, infections, or toxin exposure, the lifespan of RBCs is significantly shortened, resulting in hemolysis [7, 8]. Hemolysis patients may experience a sharp decline in hemoglobin levels, potentially progressing to severe hypoxic shock in extreme cases [9]. Additionally, hemolytic jaundice arises from the extensive breakdown of RBCs, which generates excessive unconjugated bilirubin (indirect bilirubin). When this exceeds the liver's metabolic capacity, bilirubin accumulates in the blood, causing jaundice [10].

The gut microbiota, a crucial component of the body’s immune and endocrine systems, plays a vital role in numerous physiological processes, including infection resistance, growth promotion, metabolism, and immune system regulation [11]. Recent studies have highlighted that an imbalance in the gut microbiota is a significant pathogenic factor in neonatal hyperbilirubinemia, with multiple investigations confirming its involvement in bilirubin metabolism [12, 13]. The research demonstrates that conjugated bilirubin undergoes two primary transformations facilitated by the gut microbiota. Firstly, it can be converted into urobilinogen through the removal of the glucuronic acid group, subsequently being excreted via feces. Secondly, it can be hydrolyzed into unconjugated bilirubin within the intestinal lumen. The latter form is reabsorbed across the intestinal wall, further metabolized, and re-enters the enterohepatic circulation [14]. Consequently, any imbalance in the gut microbiota can significantly impact bilirubin metabolism. Currently, there is a paucity of studies examining the gut microbiota of neonates with hemolytic jaundice. This study aims to investigate the composition and features of the gut microbiota in neonatal hemolytic jaundice, thereby providing potential biomarkers for the diagnosis of this condition.

## Materials and methods

### Study population

This is a single-center, prospective cohort study conducted at Suzhou Hospital of Anhui Medical University. We enrolled 70 neonates with hemolytic jaundice and 21 healthy neonates hospitalized in the Neonatal Department from March to September 2024 as our research subjects. The control group comprised healthy newborns who were matched in terms of age and gender with the population of hemolytic jaundice, and newborns with gastrointestinal or infectious diseases were excluded. Inclusion criteria for neonates with hemolytic jaundice: (1) meeting the diagnostic criteria for neonatal jaundice [15, 16]; (2) diagnosis of hemolytic disease of the newborn aligns with the"Expert Consensus on Laboratory Testing of Fetal and Neonatal Hemolytic Disease"established by the Immunohematology Professional Committee of the Chinese Blood Transfusion Association in 2021; (3) full-term newborn; (4) neonatal age at enrollment does not exceed 7 days; (5) the mother is in good health and free from gestational complications. Exclusion criteria: (1) presence or combination of hereditary, metabolic, or gastrointestinal diseases; (2) presence of congenital abnormalities or birth defects; (3) presence of severe organic diseases, including cardiovascular diseases, pneumonia, or abnormal renal function. (4) use of antibiotics, probiotics, prebiotics, synbiotics, or related preparations is observed; (5) during pregnancy, the mother used probiotics, prebiotics, synbiotics, or related preparations. This study was approved by the Ethics Committee of Suzhou Hospital of Anhui Medical University (No. C2024029). Written informed consent was obtained from the parents or legal guardians of all participating children.

### Fecal samples and clinical data collection

To minimize the potential influence of delivery mode on gut microbiota research outcomes, for vaginally delivered newborns, no less than 1 g of fecal samples should be collected during their first defecation upon hospital admission. In contrast, for cesarean-section-delivered newborns, no less than 1 g of fecal samples should be collected during their first defecation after one week of breastfeeding post-birth. The samples were preserved in the preservation fluid tubes supplied by Dian Diagnostics Group Co., Ltd. (Hangzhou, China) and immediately placed in an ice box. Subsequently, the samples were transported to the laboratory within 30 min and stored at −80 °C. Demographic and clinical data were systematically gathered from the electronic medical record system. Specifically, the following data points were collected: neonatal birth weight, maternal and neonatal age, gender, gestational age, mode of delivery, antibiotic usage, feeding status, maternal weight at delivery, maternal gestational age, length of hospital stay, duration of antibiotic treatment, vitro fertilization, neonatal asphyxia, umbilical cord around the neck (UCAN), and laboratory test results. All blood samples collected from pediatric patients upon admission were transferred to test tubes, centrifuged at 3,000 g for 15 min, and immediately stored at −80 °C. Subsequently, the following parameters were measured sequentially: C-reactive protein (CRP), white blood cell count (WBC), alanine transaminase (ALT), aspartate transaminase (AST), procalcitonin (PCT), neutrophil count (NEU), total bilirubin (TBIL), and direct bilirubin (DBIL).

### DNA extraction and 16S rRNA gene high-throughput sequencing

Genomic DNA from fecal samples was extracted using the PowerMax DNA extraction kit (MoBio Laboratories, Carlsbad, CA, USA) following the manufacturer's instructions. The extracted DNA was stored at −20 °C. The concentration of the extracted DNA was measured using a NanoDrop ND-1000 spectrophotometer (Thermo Fisher Scientific, Waltham, MA, USA), and the integrity of the samples was assessed by 2% agarose gel electrophoresis. The extracted DNA was deemed qualified if the A260/280 ratio from the spectrophotometer was within the range of 1.8 to 2.0 and a distinct main band was observed in the agarose gel electrophoresis.

The V4 region of the 16S rRNA gene was amplified via PCR using the primers F (5’-GTGCCAGCAGCCGCGGTAA-3’) and R (5’-GGACTACCAGGGTTTCTAAT-3’). The resulting PCR products were purified and quantified with the EasyPure Quick Gel Extraction Kit (TransGen, Beijing, China) and the PicoGreen dsDNA Assay Kit (Invitrogen, Carlsbad, CA, USA). The sequencing was performed on the Illumina HiSeq 4000 platform with a paired-end 2 × 150 bp configuration. After acquiring the raw data in FASTQ format, the barcode and primer sequences were removed. The reads from each sample were assembled using VSEARCH v2.4.4. Subsequently, the initial sequences were filtered using QIIME v1.8.0 (http://qiime.org/) to eliminate low-quality sequences, resulting in the final set of effective tags. The exclusion criteria are as follows: (1) Sequences shorter than 150 base pairs; (2) Sequences with an average quality score below 20; (3) Sequences contaminated by adapter sequences; (4) Sequences containing single nucleotide repeats longer than 8 base pairs; (5) Chimeric sequences; (6) Sequences containing ambiguous nucleotides.

With a 97% similarity threshold, tags exhibiting over 97% similarity were clustered into operational taxonomic units (OTUs) using the USEARCH software. Subsequently, species annotation of the OTU sequences was performed using the Vsearch 2.4.4 software and the SILVA128 database to generate the final OTU list. The microbial community composition was statistically analyzed at each taxonomic level before documenting the abundance and classification of all OTUs in each sample. Any OTUs representing less than 0.001% of the total sequences across all samples were excluded.

### Bioinformatics analysis

The QIIME software was utilized to compute the alpha diversity index and Good's coverage, as well as to generate the ranked abundance curve. Alpha diversity primarily evaluates species diversity through two metrics: abundance indices (Chao1, ACE) and diversity indices (Shannon, Simpson). The"Venn Diagram"function in the R package was employed to create a visual representation of the Venn diagram, illustrating the abundance and similarity of the gut microbiota composition across different groups. Principal Coordinate Analysis (PCoA) of beta diversity (Bray–Curtis distance), generated using QIIME2 software, was utilized to assess the differences in the composition and structure of the gut microbiota among the groups. Using the “Function betadisper” from the R package, an ANOSIM test was performed to assess the significance of differences between groups. Additionally, linear discriminant analysis (LDA) effect size (LEfSe) was employed to identify species with significant differences in relative abundance between groups (function ldamarker, R package v3.2.0). Utilizing the GO and KEGG databases, the PICRUSt2 software was employed to investigate the functional disparities of microorganisms across different groups. Additionally, the metagenomeSeq software was utilized to examine the metabolic functions of differential gut microbiota.

### Statistical analysis

Statistical analysis was conducted using SPSS (version 24.0). All measurement data followed a normal distribution and were presented as the mean ± standard deviation. Comparisons among the two groups were performed using an t-tests. Count data were reported as [n (%)], and group comparisons were analyzed using the χ^2^ test. A *p*-value < 0.05 was deemed statistically significant for all analyses.

## Results

### Baseline information

A total of 70 patients with hemolytic jaundice were enrolled in the study. A total of 8 patients were excluded: 5 due to poor compliance during feces collection, 2 with pneumonia, and 1 with severe diarrhea. Consequently, 62 eligible neonates with hemolytic jaundice were included in the Treatment group, comprising 27 males and 35 females. The mean age was (2.03 ± 0.89) days, with weights ranging from 2600 to 4650 g. Twenty-one healthy newborns were recruited as the Control group during the same period. One sample of fecal collection failed to meet the inclusion criteria. Consequently, 20 eligible newborns were included in the study, comprising 8 males and 12 females, with an average age of (1.86 ± 0.68) days, a body weight range of 2750 to 4100 g, and maternal ages ranging from 24 to 36 years. No significant differences were observed in age, gender, or birth weight between the hemolytic jaundice group and the control group.

The clinical characteristics of the newborns are presented in Table 1. No significant differences were observed between the two groups regarding age, gender, gestational age, birth weight, delivery, maternal age, maternal weight, exclusive breastfeeding, vitro fertilization, neonatal asphyxia, UCAN, PCT, WBC, and NEU. However, the levels of CRP, AST, TBIL, and DBIL were significantly higher in the Treatment group compared to the Control group.

**Table 1:** Baseline characteristics of study populations

Variables	Treatment group (*n* = 62)	Control group (*n* = 20)	*p-value*
**Demographic**			
Age, days	2.03 ± 0.89	1.86 ± 0.68	0.677
Male	27 (43.55)	8 (40.00)	0.686
Gestational age (week)	38.95 ± 1.11	39.35 ± 1.13	0.874
Birth weight (g)	3392.26 ± 238.60	3498.00 ± 341.09	0.532
Delivery			0.122
Cesarean section	19 (30.65)	10 (50.00)	
Vaginal	43 (69.35)	10 (50.00)	
Maternal age (year)	30.29 ± 3.44	30.60 ± 3.06	0.582
Maternal weight (kg)	74.26 ± 10.78	74.20 ± 14.50	0.819
Exclusive breastfeeding			0.393
Yes	2 (3.23)	0 (0.00)	
No	60 (96.77)	20 (100.00)	
Vitro fertilization			0.568
Yes	1 (1.61)	0 (0.00)	
No	61 (98.39)	20 (100.00)	
UCAN			0.190
Yes	4 (6.45)	0 (0.00)	
No	58 (93.55)	20 (100.00)	
Neonatal asphyxia			0.190
Yes	1 (1.61)	0 (0.00)	
No	61 (98.39)	20 (100.00)	
**Clinical variables**			
ALT, U/L	15.264 ± 2.33	18.05 ± 4.03	0.672
AST, U/L	65.68 ± 7.07	20.43 ± 8.17	0.006
CRP, mg/L	5.74 ± 1.26	2.37 ± 1.51	0.032
PCT, ng/mL	1.28 ± 0.93	1.09 ± 0.86	0.872
WBC, 10^9^/L	14.56 ± 2.03	15.47 ± 5.92	0.562
NEU, %	60.05 ± 5.16	63.60 ± 9.79	0.598
TBIL, μmol/L	223.76 ± 52.09	97. 99 ± 26.68	< 0.001
DBIL, μmol/L	33.76 ± 2.24	7.00 ± 1.23	< 0.001

Data presented as n (%), mean ± standard deviation

*ALT* alanine aminotransferase, *AST* aspartate transaminase, *CRP* C-reaction protein, *PCT* procalcitonin, *WBC* white blood cell, *UCAN* umbilical cord around the neck, *NEU* Neutrophil, *TBIL* total bilirubin, *DBIL* direct bilirubin

### Gut microbiota diversity analysis

**Fig. 1:**
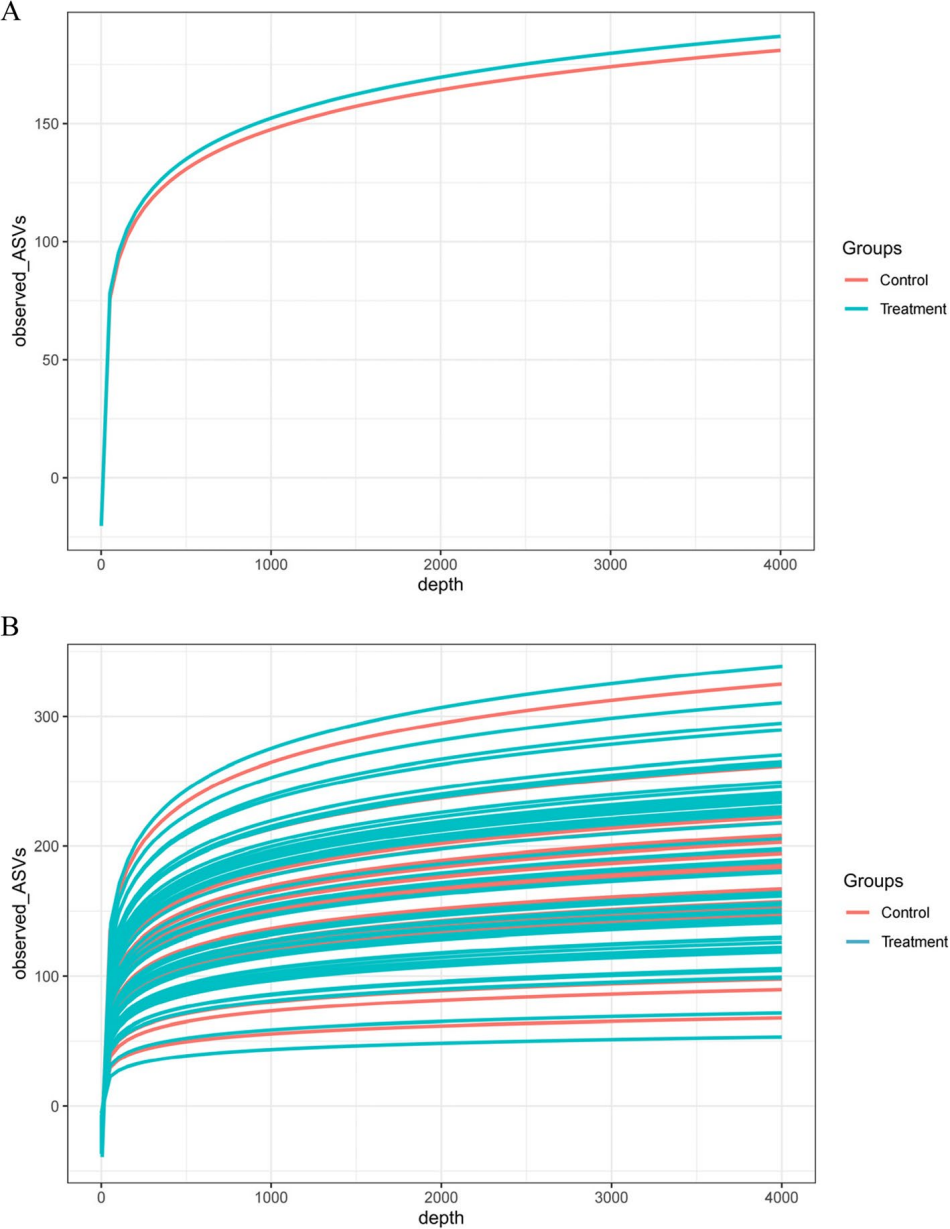
Alpha rarefaction analysis of operational taxonomic units. **A** Alpha rarefaction analysis of operational taxonomic units in group. **B** Alpha rarefaction analysis of operational taxonomic units among the samples

To investigate the alterations in the gut microbiota of neonates with hemolytic jaundice, 16S rRNA sequencing was conducted on fecal samples from both groups. A total of 1,160 ASVs were identified across the samples, and the good's coverage indices for the observed OTUs in all samples exceeded 99%, confirming the reliability of the sequencing results. The Alpha rarefaction analysis of OTU between groups and among samples demonstrated that the sequencing depth was adequate to meet the requirements and accurately represent the richness of the gut microbiota, thereby minimizing potential biases due to varying sample sizes between groups (Fig. 1).

**Fig. 2:**
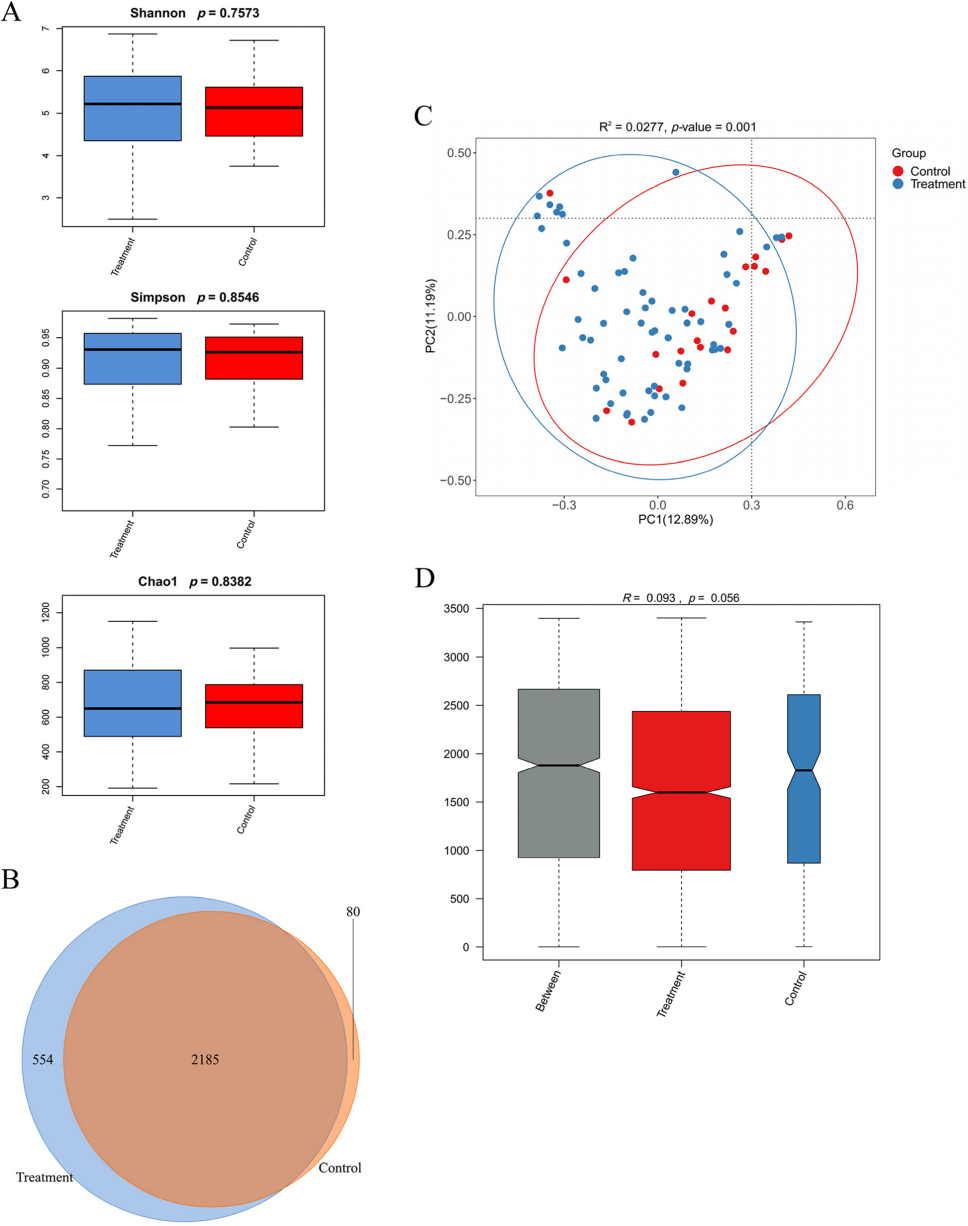
Gut microbiota diversity analysis. **A** Alpha diversity index between the two groups. **B** Venn diagram among the two groups. **C** Principal coordinate analysis (PCoA) based on weighted UniFrac distances in two groups. **D** Beta diversity Anosim analysis among the two groups

There was no significant difference in the Alpha diversity index between the two groups (Fig. 2A), with the *p*-values for the Shannon, Simpson, and Chao1 indices being 0.757, 0.855, and 0.838, respectively. The Venn diagram revealed that a total of 2819 OTUs were identified, with 2739 in the treatment group and 2265 in the control group. Notably, 2185 OTUs were shared between the two groups (Fig. 2B). PCoA of beta diversity reveals significant differences in gut microbiota composition between the two groups (Fig. 2C, *p* < 0.001). This finding is further supported by ANOSIM analysis (*R* = 0.058, *p* = 0.017, Fig. 2D).

**Fig. 3:**
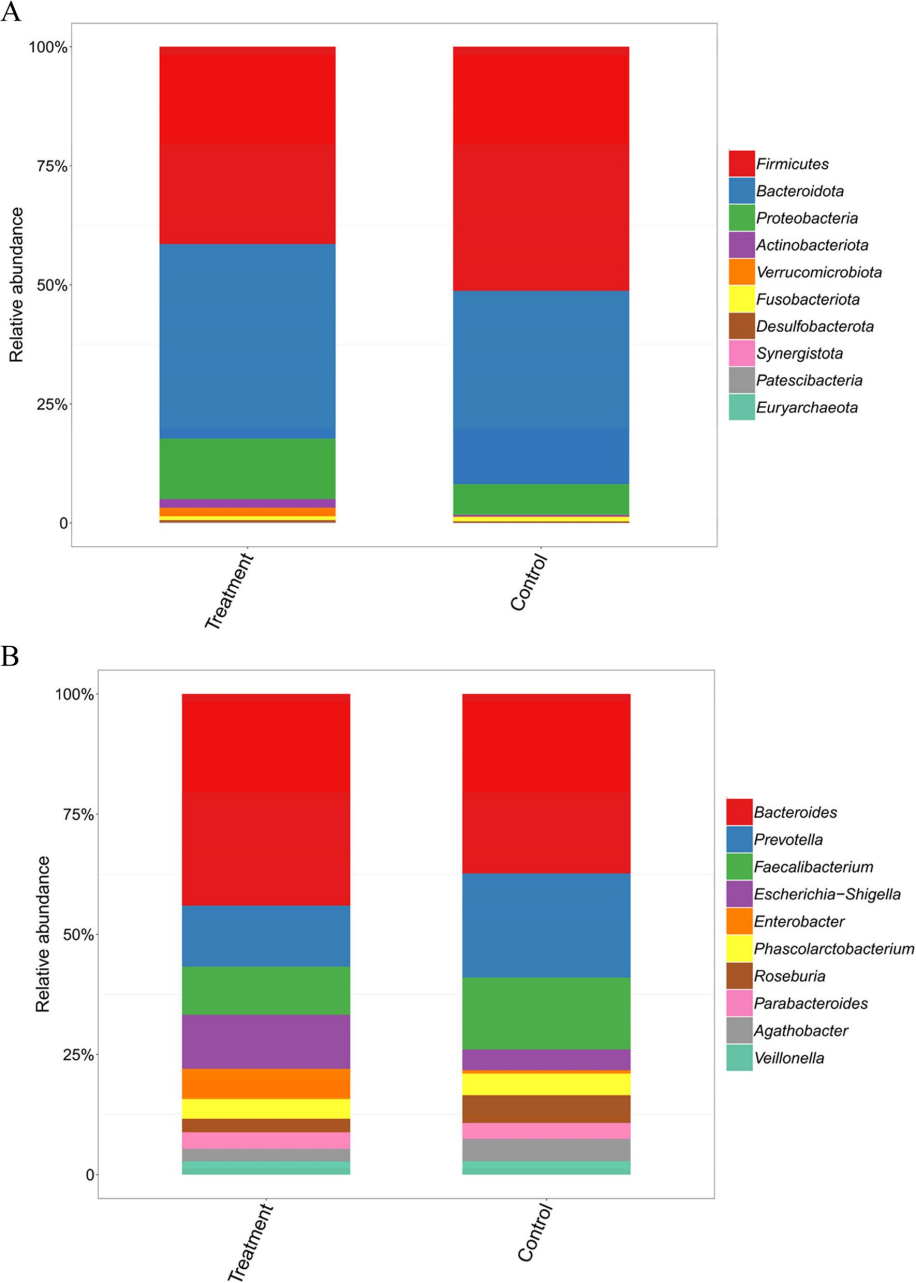
Taxonomic features of gut microbiota among the two groups. **A** Relative abundances of bacteria at the phylum level. **B** Relative abundances of bacteria at the genus level

Taxonomic profiles at different levels are depicted in Fig. 3. Across all groups, the predominant phyla are *Firmicutes*, *Bacteroidota*, *Proteobacteria*, *Actinobacteriota*, and *Verrucomicrobiota*, with respective *p*-values for the difference tests among the three groups being 0.017, 0.691, 0.009, 0.029, and 0.063 (Fig. 3A). At the genus level, *Bacteroides*, *Prevotella*, *Faecalibacterium*, *Escherichia-Shigella*, *Enterobacter*, *Phascolarctobacterium*, *Roseburia*, *Parabacteroides*, *Agathobacter*, and *Veillonella* are the primary dominant genera (Fig. 3B).

### Differential microbiota between the two group

**Fig. 4:**
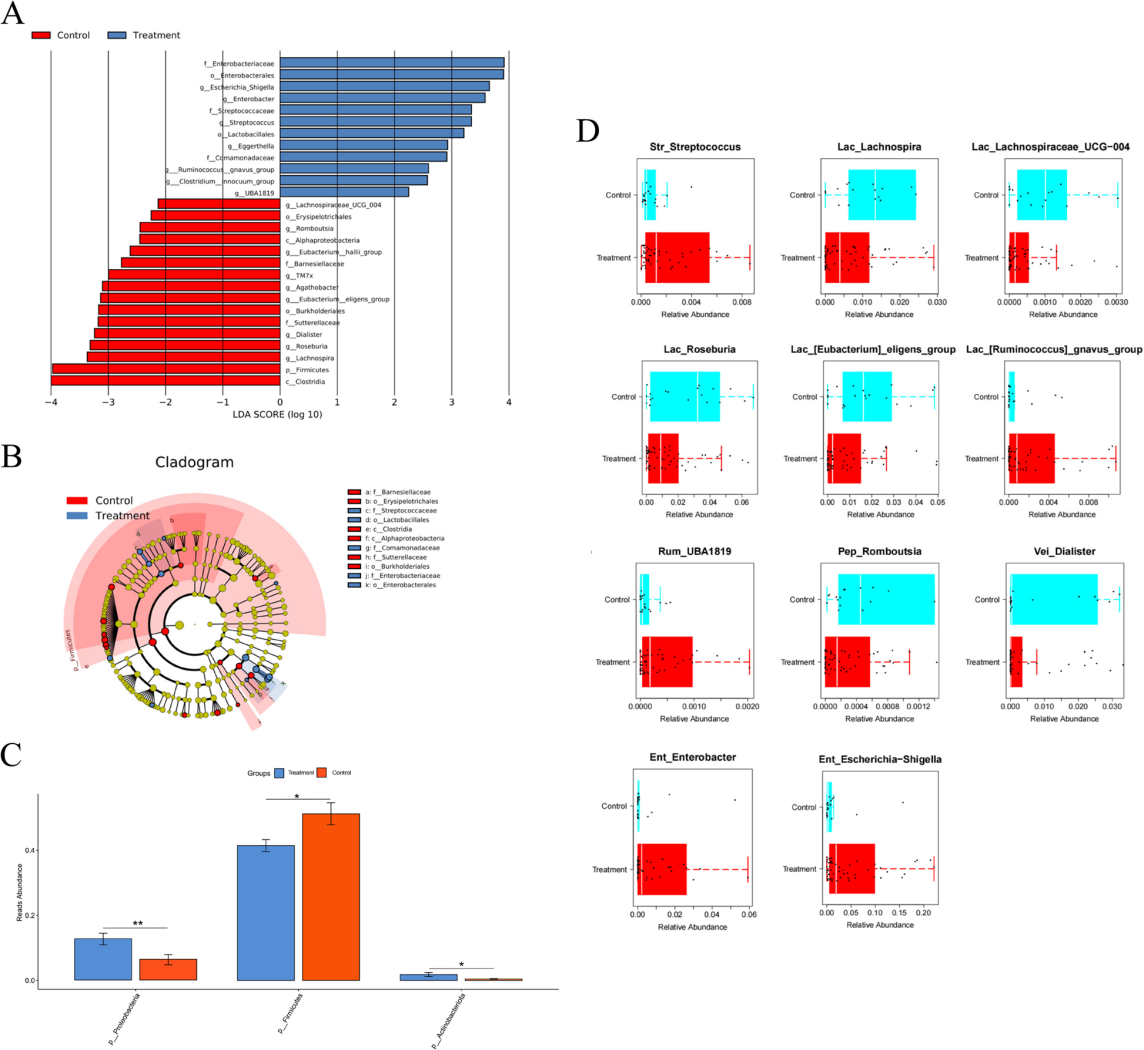
Differential microbiota analysis. **A** LDA analysis results. **B** LEfSe analysis clustering tree. ASV, amplicon sequence variants; p, c, o, f, and g represent phylum, class, order, family and genus, respectively. **C** Relative abundance of the most significant phylum. **D** Relative abundance of the most significant genus. *, *p* < 0.05; **, *p* < 0.01

We employed LEfSe to identify differential gut microbiota between the two groups (Fig. 4A). At the genus level, 7 genera were significantly enriched in the Treatment group, such as *Escherichia-Shigella*, *Enterobacter*, *Streptococcus*, *Eggerthella*, *Ruminococcus*, *Clostridium*, and *UBA1819*. In contrast, 9 genera were significantly enriched in the Control group, such as *Lachnospiraceae UCG-004*, *Romboutsia*, *Eubacterium hallii group*, *TM7X*, *Agathobacter*, *Eubacterium eligens group*, *Dialister*, *Roseburia*, and *Lachnospira*. At the phylum level, *Firmicutes* was significantly enriched in the Control group. The LEfSe cladogram further illustrated distributional differences of gut microbiota (Fig. 4B).

We analyzed the differences in the intestinal microbiota composition at both the phylum and genus levels. At the phylum level, compared to the Control group, the Treatment group exhibited an enrichment of *Proteobacteria* and *Actinobacteriota*, whereas *Firmicutes* showed a significant decrease (Fig. 4C). At the genus level, the relative abundances of *Streptococcus*, *Escherichia-Shigella*, *Enterobacter*, *UBA1819*, and *Ruminococcus gnavus group* in the Treatment group were significantly higher compared to the Control group. Conversely, the relative abundances of *Lachnospira*, *Lachnospiraceae UCG-004*, *Roseburia*, *Eubacterium eligens group*, *Romboutsia*, and *Dialister* were significantly lower in the Treatment group compared to the Control group (Fig. 4D).

### Gut microbial function analysis

**Fig. 5:**
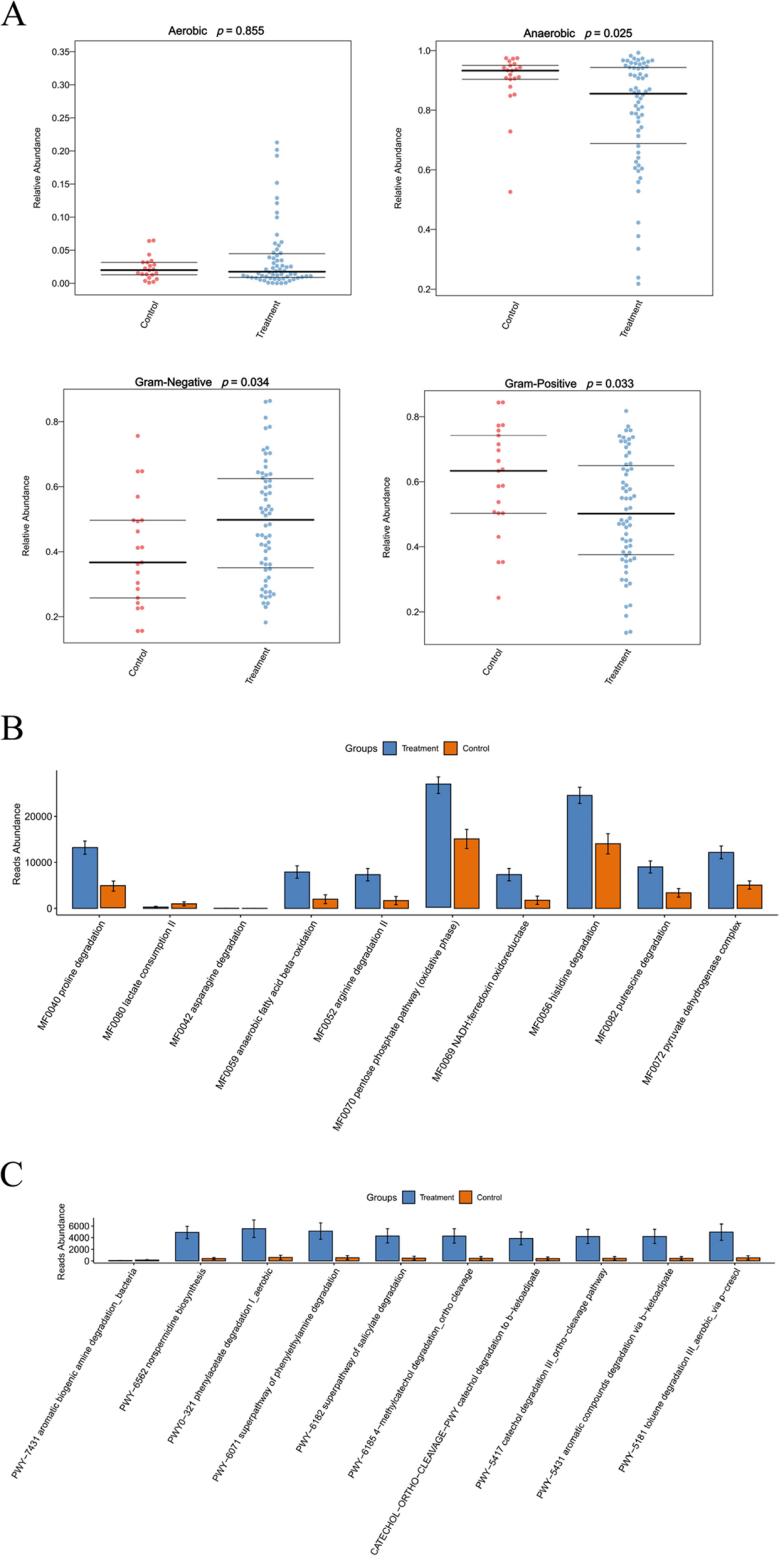
Gut microbiological function analysis. **A** Predicted relative abundance of aerobic bacteria, anaerobic bacteria, Gram-negative bacteria, and Gram-positive bacteria. Statistical analysis was performed by pairwise Mann–Whitney-Wilcoxon test. **B** Metabolic pathways based on PICRUSt2. **C** METACYC analysis based on PICRUSt2

We analyzed and compared the abundances of aerobic bacteria, anaerobic bacteria, Gram-positive bacteria, and Gram-negative bacteria in the gut microbiota between the two groups (Fig. 5A). The results indicated that the relative abundance of Gram-positive bacteria in the Treatment group was significantly higher than in the Control group (*p* < 0.05), whereas the relative abundances of anaerobic and Gram-negative bacteria in the Treatment group were significantly lower than in the Control group (*p* < 0.05).

We conducted an analysis of the metabolic pathways of the gut microbiota using PICRUSt2 (Fig. 5B). Our findings revealed that the metabolic activities of proline degradation (MF0040), anaerobic fatty acid beta-oxidation (MF0059), arginine degradation II (MF0052), pentose phosphate pathway (MF0070), NADH:ferredoxin oxidoreductase (MF0069), histidine degradation (MF0056), putrescine degradation (MF0082), and pyruvate dehydrogenase complex (MF0072) were significantly elevated in the Treatment group (*p* < 0.05), whereas the metabolic activity of lactate consumption II (MF0080) was significantly reduced (*p* < 0.05). Furthermore, METACYC analysis revealed a significant increase in the metabolic activities of norspermidine biosynthesis (PWY-6562), aerobic phenylacetate degradation (PWY0-321), phenylethylamine degradation superpathway (PWY-6071), salicylate degradation superpathway (PWY-6182), 4-methylcatechol degradation via ortho cleavage (PWY-6185), catechol degradation to β-ketoadipate (CATECHOL-ORTHO-CLEAVAGE-PWY), catechol degradation III via ortho cleavage (PWY-5417), aromatic compounds degradation via β-ketoadipate (PWY-5431), and aerobic toluene degradation via p-cresol (PWY-5181) in the Treatment group, as shown in Fig. 5C (*p* < 0.05).

### Differential bacteria of function analysis

**Fig. 6:**
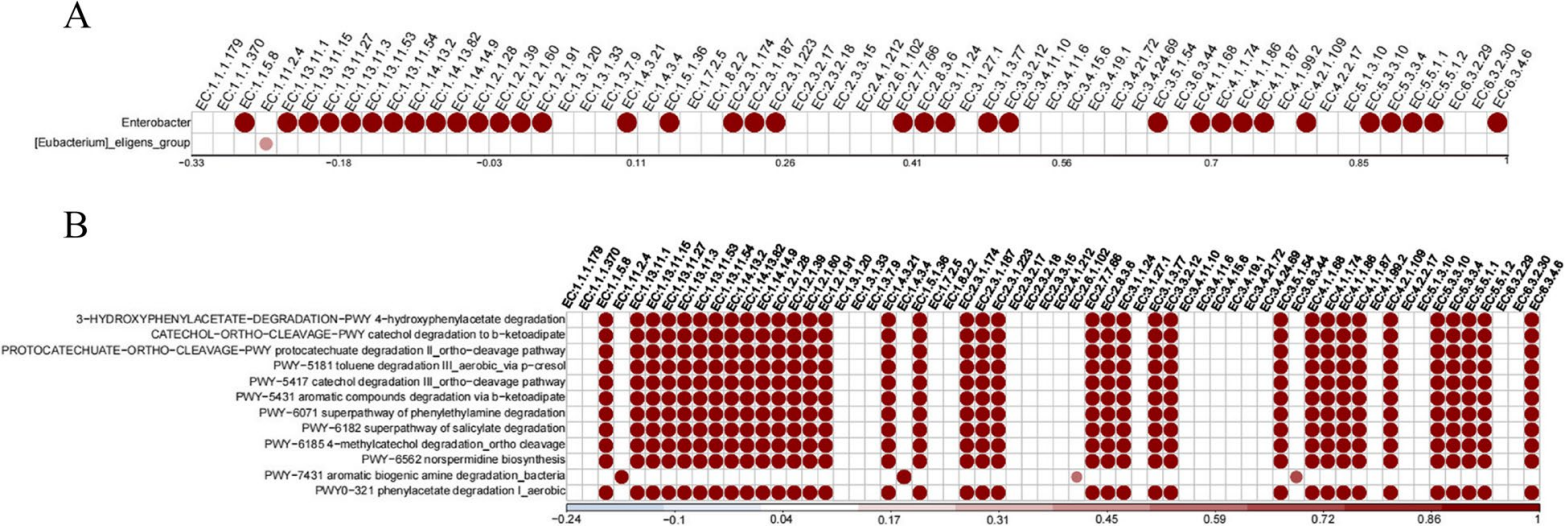
Differential bacteria of function analysis. **A** Correlation between EC modules and differential microbiota. **B** Correlation between EC modules and METACYC metabolic modules

The correlation between EC modules and differential microbiota is illustrated in Fig. 6A. Correlation analysis revealed that *Enterobacter* exhibited a positive correlation with the secretion of various enzymes, including oxidases, reductases, transferases, hydrolases, isomerases, and lyases. Further correlation analysis between EC and METACYC metabolic modules demonstrated that these enzymes were positively correlated with the metabolic levels of several pathways: 4-hydroxyphenylacetate degradation (3-HYDROXYPHENYLACETATE-DEGRADATION-PWY), catechol degradation to β-ketoadipate (CATECHOL-ORTHO-CLEAVAGE-PWY), protocatechuate degradation II_ortho-cleavage pathway (PROTOCATECHUATE-ORTHO-CLEAVAGE-PWY), toluene degradation III_aerobic_via p-cresol (PWY-5181), catechol degradation III_ortho-cleavage pathway (PWY-5417), aromatic compounds degradation via β-ketoadipate (PWY-5431), superpathway of phenylethylamine degradation (PWY-6071), superpathway of salicylate degradation (PWY-6182), 4-methylcatechol degradation_ortho cleavage (PWY-6185), norspermidine biosynthesis (PWY-6562), aromatic biogenic amine degradation_bacteria (PWY-7431), and phenylacetate degradation I_aerobic (PWY-0321). Consequently, we associated *Enterobacter* with these metabolic pathways (Fig. 6B). Notably, these metabolic pathways were highly consistent with the differential METACYC metabolic profiles observed between groups.

## Discussion

The dysbiosis of the gut microbiota results in disturbances in bilirubin metabolism, a finding supported by both animal and human studies [17, 18]. However, the relationship between the imbalance of the gut microbiota in hemolytic disease of newborn and the development of jaundice remains uncertain. Therefore, this study aims to investigate the composition and features of the gut microbiota in newborns with hemolytic jaundice, with the goal of identifying potential biomarkers for the diagnosis of hemolytic jaundice. In this study, we performed 16S rRNA gene sequencing on fecal samples from 62 neonates with hemolytic jaundice and 20 healthy neonates, and further examined the differences in gut microbiota between the two groups. The good's coverage and alpha rarefaction analysis of all samples demonstrated that the sequencing depth, species richness, and evenness of all samples satisfied the prerequisites for subsequent analysis. The results of the alpha diversity analysis indicated no significant difference in the richness and diversity of the gut microbiota between the two groups. A review of existing literature revealed that the alpha diversity of the gut microbiota in neonates with hemolytic jaundice remains largely unchanged. These findings are generally consistent with the conclusions drawn from prior studies [19, 20]. Beta diversity analysis revealed a significant difference in the composition of the gut microbiota between the two groups. A review of previous studies indicated that findings on the beta diversity of the gut microbiota in jaundiced newborns have been inconsistent. Zhou et al. reported a significant difference in the beta diversity of the gut microbiota between jaundiced neonates and healthy neonates [21]. Conversely, Dong et al. found no significant difference in the beta diversity of the gut microbiota between jaundiced neonates and non-jaundiced neonates [22]. The discrepancies from the previously reported results can be attributed to several factors, including variations in disease classification, geographical location, and dietary patterns [23–25]. Firstly, this study was conducted in the eastern region of China, whereas most prior studies have primarily focused on foreign countries or different regions within China. Consequently, it is plausible that regional and environmental factors influence the gut microbiota. Secondly, this study specifically examined hemolytic jaundice, while the majority of existing research has generally centered on jaundiced newborns without further categorizing the subjects.

At the phylum level, we observed an increase in the relative abundances of *Proteobacteria* and *Actinobacteriota* in the gut microbiota of neonates with hemolytic jaundice, while the relative abundance of *Firmicutes* exhibited a downward trend. *Proteobacteria* encompasses a substantial number of potentially pathogenic microorganisms, and an abnormal increase or imbalance in their relative abundance may be closely linked to various disease states. Research has demonstrated that *Enterobacteriaceae*, which belong to the *Proteobacteria*, are associated with the development of inflammatory bowel disease (IBD) [26]. Furthermore, certain members within the *Proteobacteria* have also been identified as having potential connections to obesity and other metabolic disorders [27]. *Actinobacteriota* is not a predominant constituent of the gut microbiota, and an elevated relative abundance of this phylum may be associated with intestinal dysbiosis. Certain members of the *Actinobacteriota* possess the capability to produce enzymes and participate in the metabolic processes involving bilirubin. These bacteria might lower serum bilirubin levels by enhancing the enterohepatic circulation of bilirubin [28]. The observed increase in *Actinobacteriota* within the gut microbiota of neonates with hemolytic jaundice could suggest that the body is attempting to compensate for aberrant bilirubin metabolism through adjustments in the intestinal microecology. *Firmicutes* constitutes a predominant component of the gut microbiota and is crucial for maintaining intestinal health and optimizing digestive and absorptive functions [29]. A decrease in the abundance of *Firmicutes* in the gut microbiota of neonates with hemolytic jaundice could impair the efficiency of bilirubin metabolism and excretion, potentially exacerbating jaundice symptoms. Furthermore, this alteration might weaken immune function and elevate the risk of neonatal infections and other conditions [30].

To elucidate the alterations in gut microbiota at the genus level, we performed LEfSe analysis and differential abundance testing. Our findings revealed that the relative abundances of *Streptococcus*, *Escherichia-Shigella*, *Enterobacter*, *UBA1819*, and *Ruminococcus gnavus group* were significantly higher in the Treatment group, whereas the relative abundances of *Lachnospira*, *Lachnospiraceae UCG-004*, *Roseburia*, *Eubacterium eligens group*, *Romboutsia*, and *Dialister* were notably lower. A study conducted in western China demonstrated that, at the genus level, *Escherichia-Shigella* and *Enterobacteriaceae* were predominant. Neonates with breast milk jaundice (BMJ) exhibited a higher abundance of *Streptococcus* compared to the healthy control group, whereas the abundance of *Enterococcus* was significantly lower in the BMJ group than in the healthy control group [31]. Another study conducted in southern China also reported a significant increase in the abundance of *Enterobacter* and *Escherichia-Shigella* in jaundiced newborns, alongside a notable decrease in *Lactobacillus* abundance [32]. Notably, both studies consistently observed a significant rise in the abundance of *Enterobacter* and *Escherichia-Shigella* in jaundiced neonates.

To further investigate the correlation between the gut microbiota and bilirubin metabolism in neonates with hemolytic jaundice, we employed PICRUSt for predictive functional analysis and correlation analysis. It is noteworthy that the correlation analysis between EC modules, METACYC metabolic modules, and differential microbiota revealed that *Enterobacter*, through the secretion of numerous enzymes, significantly influences the metabolic pathways involved in the degradation of catechol to β-ketoadipate (CATECHOL-ORTHO-CLEAVAGE-PWY), aerobic toluene degradation via p-cresol (PWY-5181), catechol degradation via ortho-cleavage (PWY-5417), degradation of aromatic compounds via β-ketoadipate (PWY-5431), the superpathway of phenylethylamine degradation (PWY-6071), the superpathway of salicylate degradation (PWY-6182), 4-methylcatechol degradation via ortho-cleavage (PWY-6185), and norspermidine biosynthesis (PWY-6562) in neonates with hemolytic jaundice. These metabolic pathways are intricately linked to liver function. These metabolic pathways are intricately linked to liver function. The liver plays a pivotal role in modulating the progression of hemolytic diseases and contributes to the regulation of hemolysis through diverse mechanisms. First, the excessive destruction of red blood cells releases substantial amounts of hemoglobin into the bloodstream. The liver synthesizes specific proteins that bind free hemoglobin and heme, facilitating their degradation and metabolism. Second, the liver metabolizes heme into bilirubin and excretes it via bile. Excessive accumulation of bilirubin can lead to jaundice. Furthermore, by regulating iron absorption and release, the liver influences the hematopoietic function of the bone marrow, indirectly modulating red blood cell production. Lastly, in immune-mediated hemolytic diseases, the liver clears red blood cells marked by antibodies or complement proteins, thereby mitigating hemolysis. Therefore, we hypothesize that the increased abundance of Enterobacter in the intestine may disrupt the liver's metabolic function, impair its capacity to process and clear bilirubin from the blood, leading to bilirubin accumulation in the blood and ultimately contributing to disease onset.

Our research acknowledges several limitations that warrant consideration. Firstly, the notable variations in the gut microbiota of the study population are largely attributable to the distinct geographical locations and dietary practices across different regions. The subjects in this study predominantly originated from the Anhui Province, which inherently constrains the generalizability and applicability of our findings. Secondly, the relatively modest sample size may further compromise the external validity of our research outcomes. Meanwhile, this study was unable to fully exclude the potential influence of the neonatal birth mode on the gut microbiota. Lastly, additional factors pertinent to neonates with hemolytic jaundice, including their intestinal nutritional status and exposure to antibiotics, were not comprehensively addressed in this study. Subsequently, our research will primarily concentrate on nursing intervention strategies in the treatment of neonatal hemolytic jaundice, aiming to enhance liver metabolic function through the regulation of intestinal flora imbalance using probiotics.

## Conclusion

Our research findings indicate that the ecological imbalance of *Enterobacter* is associated with the hepatic metabolic function of neonates suffering from hemolytic jaundice. We hypothesize that *Enterobacter* is intricately linked to multiple metabolic pathways in the liver via the secretion of a wide array of enzymes, as mediated by the gut-liver axis metabolic pathway. The alteration in the relative abundance of *Enterobacter* indirectly indicates the metabolic state of the liver and the progression of disease in neonatal hemolytic jaundice. Consequently, *Enterobacter* may serve as a promising diagnostic marker within the intestinal flora for assessing liver metabolic disorders associated with hemolytic jaundice.

## Data Availability

The raw microbiome data have been archived in the NCBI Sequence Read Archive (SRA) repository under the accession number PRJNA1207199.

## Abbreviations

LEfSe: Linear discriminant analysis effect size
NEU: Neutrophil count
TBIL: Total bilirubin
ALT: Alanine transaminase
AST: Aspartate transaminase
CRP: C-reaction protein
WBC: White blood cell count
DBIL: Direct bilirubin
PCT: Procalcitonin
QIIME: Quantitative insights into microbial ecology
OTU: Operational taxonomic units
PCoA: Principal coordinate analysis
ASV: Amplicon sequence variant
ANOSIM: Analysis of similarities
LDA: Linear discriminant analysis
KEGG: Kyoto encyclopedia of genes and genomes
UCAN: Umbilical cord around the neck
IBD: Inflammatory bowel disease
BMJ: Breast milk jaundice
